# Genome sequence of the *Lotus corniculatus* microsymbiont *Mesorhizobium loti* strain R88B

**DOI:** 10.1186/1944-3277-9-3

**Published:** 2014-12-08

**Authors:** Wayne Reeve, John Sullivan, Clive Ronson, Rui Tian, Lambert Bräu, Karen Davenport, Lynne Goodwin, Patrick Chain, Tanja Woyke, Elizabeth Lobos, Marcel Huntemann, Amrita Pati, Konstantinos Mavromatis, Victor Markowitz, Natalia Ivanova, Nikos Kyrpides

**Affiliations:** 1 Centre for Rhizobium Studies, Murdoch University, Western Australia, Australia; 2 Department of Microbiology and Immunology, University of Otago, Dunedin, New Zealand; 3 School of Life and Environmental Sciences, Deakin University, Victoria, Australia; 4 Los Alamos National Laboratory, Bioscience Division, Los Alamos, New Mexico, USA; 5 DOE Joint Genome Institute, Walnut Creek, CA, USA; 6 Biological Data Management and Technology Center, Lawrence Berkeley National Laboratory, Berkeley, CA, USA; 7 Department of Biological Sciences, King Abdulaziz University, Jeddah, Saudi Arabia

**Keywords:** Root-nodule bacteria, Nitrogen fixation, Symbiosis, *Alphaproteobacteria*

## Abstract

*Mesorhizobium loti* strain R88B was isolated in 1993 in the Rocklands range in Otago, New Zealand from a *Lotus corniculatus* root nodule. R88B is an aerobic, Gram-negative, non-spore-forming rod. This report reveals the genome of *M. loti* strain R88B contains a single scaffold of size 7,195,110 bp which encodes 6,950 protein-coding genes and 66 RNA-only encoding genes. This genome does not harbor any plasmids but contains the integrative and conjugative element ICE*Ml*Sym^R7A^, also known as the R7A symbiosis island, acquired by horizontal gene transfer in the field environment from *M. loti* strain R7A. It also contains a mobilizable genetic element ICE*Ml*adh^R88B^, that encodes a likely adhesin gene which has integrated downstream of ICE*Ml*Sym^R7A^, and three acquired loci that together allow the utilization of the siderophore ferrichrome. This rhizobial genome is one of 100 sequenced as part of the DOE Joint Genome Institute 2010 Genomic Encyclopedia for *Bacteria* and *Archaea*-Root Nodule Bacteria (GEBA-RNB) project.

## Introduction

*Mesorhizobium loti* strain R88B was first described in studies that culminated in the discovery of the *M. loti* strain R7A symbiosis island [1,2]. The research involved the characterization of genetic diversity within a population of mesorhizobia found beneath a stand of *Lotus corniculatus* located in the Rocklands range in Central Otago New Zealand. The site was established with a single inoculum strain ICMP3153 in an area lacking indigenous rhizobia capable of nodulating the plant. A group of genetically diverse mesorhizobial strains that included R88B were isolated from nodules seven years after the site was established. A field reisolate of ICMP3153 designated R7A was also isolated from the site and this strain has subsequently been used widely for molecular studies. Analysis of the diverse strains revealed that they all contained identical symbiotic DNA. Characterization of these strains led to the discovery of the 502-kb R7A symbiosis island, a mobile integrative and conjugative element that was subsequently renamed ICE*Ml*Sym^R7A^[3]. R88B contains no plasmids but ICE*Ml*Sym^R7A^ is integrated at the phe-tRNA gene [1,4]. On the basis of DNA-DNA hybridization, multi-locus enzyme electrophoresis and 16S rDNA sequence, R88B was shown to belong to the same genomic species as other symbiotic isolates and several nonsymbiotic isolates from the Rocklands site, but that strain R7A belonged to a different genomic species [5].

Examination of the genome sequence downstream of ICE*Ml*Sym^R7A^ in R88B revealed the presence of another ICE, ICE*Ml*adh^R88B^, that encoded a large (4681 amino acids) adhesin-like protein with 34 VCBS repeats and two proteins that likely comprise a Type I secretion system for the adhesin. ICE*Ml*adh^R88B^ also encoded an integrase, excisionase and *traACD* genes, indicating that the element is likely mobilizable by self-conjugative elements such as ICE*Ml*Sym^R7A^. The discovery of ICE*Ml*adh^R88B^ showed that genomic islands can integrate in tandem at the phe-tRNA locus and also indicated that mesorhizobia may gain adaptive traits by acquisition of integrated genomic islands rather than plasmids [6].

*M. loti* strain R88B was also the focus of a study that catalogued variation in the ability to utilize the siderophore ferrichrome within the diverse set of *M. loti* strains [7]. Within R88B, the functional *fhu* genes were found to be present in three co-ordinately regulated loci, each of which was independently acquired by the strain. The genes *fhu*BD that encode two of the three subunits of the ferrichrome ABC transporter were located downstream of ICE*Ml*adh^R88B^ and were absent from the previously sequenced genome of *M. loti* strain MAFF303099. This suggests that these genes may have been part of another ICE that had integrated at the phe-tRNA locus. The finding that RirA binding sites were located upstream of the loci suggests that the genes are probably subject to regulation by the iron-responsive repressor RirA, a copy of which is present in the R88B genome. The mosaic nature of the R88B *fhu* system, the variability observed in the ability of *M. loti* strains isolated from several sites in Central Otago, New Zealand to utilize ferrichrome, and the patchwork distribution of *fhu* genes in these strains suggests that these loci evolved through cycles of gene acquisition and deletion, with the positive selection pressure of an iron-poor or siderophore-rich environment being offset by the negative pressure of the Fhu receptor being a target for phage [7].

Here we present a summary classification and a set of general features for *M. loti* strain R88B together with the description of the complete genome sequence and annotation.

## Organism information

**Figure 1:**
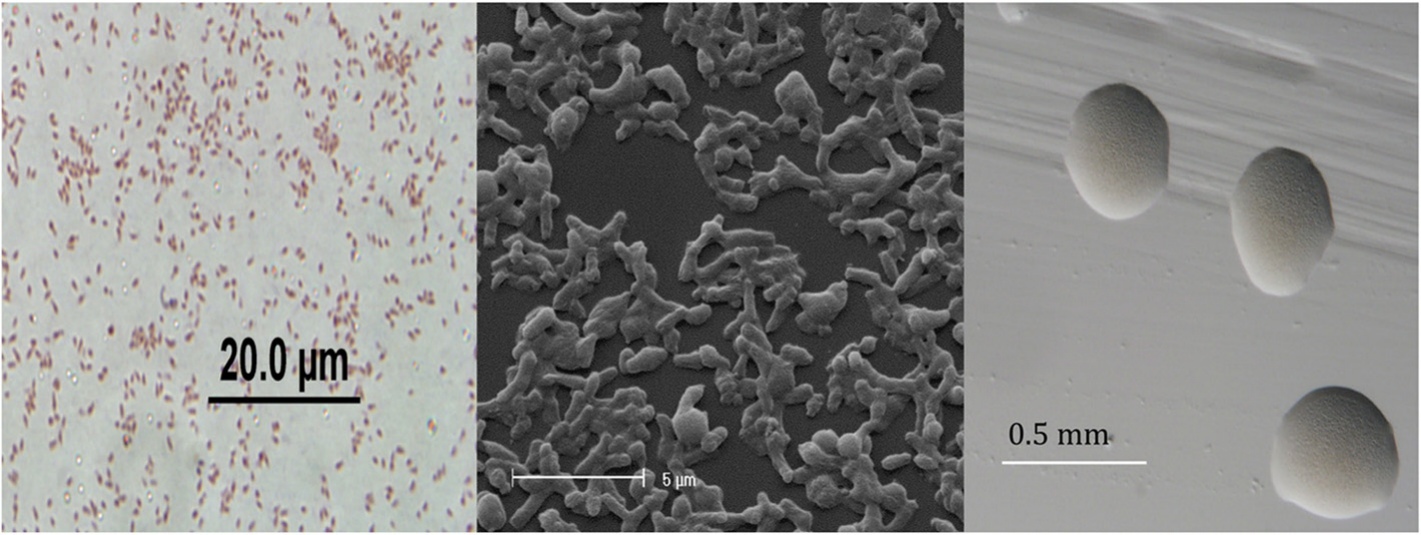
**Images of *Mesorhizobium loti *** **strain R88B from a Gram stain (Left), using scanning electron microscopy (Center) and the appearance of colony morphology on ½ LA (Right).**

*Mesorhizobium loti* strain R88B is in the order *Rhizobiales* of the class *Alphaproteobacteria*. Cells are described as non-sporulating, Gram-negative (Figure 1 Left), non-encapsulated, rods. The rod-shaped form varies in size with dimensions of 0.25–0.5 μm in width and 1–2 μm in length (Figure 1 Left and Center). They are moderately fast growing, forming 1 mm diameter colonies within 6 days and have a mean generation time of approximately 8–12 h when grown in TY broth at 28°C [1]. Colonies on G/RDM agar [8] and half strength Lupin Agar (½LA) [9] are white-opaque, slightly domed, mucoid with smooth margins (Figure 1 Right).

Strains of this organism are able to tolerate a pH range between 4 and 10. Carbon source utilization and fatty acid profiles of *M. loti* have been described previously [10-12]. Minimum Information about the Genome Sequence (MIGS) is provided in Table 1.

**Table 1:** **Classification and general features of *Mesorhizobium loti *** **strain R88B according to the MIGS recommendations**[13,14]

**MIGS ID**	**Property**	**Term**	**Evidence code**
	Current classification	Domain *Bacteria*	TAS [14]
		Phylum *Proteobacteria*	TAS [15]
		Class *Alphaproteobacteria*	TAS [16,17]
		Order *Rhizobiales*	TAS [17,18]
		Family *Phyllobacteriaceae*	TAS [17,19]
		Genus *Mesorhizobium*	TAS [11]
		Species *Mesorhizobium loti*	TAS [10,11]
		Strain R88B	TAS [1]
	Gram stain	Negative	IDA
	Cell shape	Rod	IDA
	Motility	Motile	IDA
	Sporulation	Non-sporulating	NAS
	Temperature range	Mesophile	NAS
	Optimum temperature	28°C	NAS
	Salinity	Unknown	NAS
MIGS-22	Oxygen requirement	Aerobic	TAS [10]
	Carbon source	Various	TAS [11]
	Energy source	Chemoorganotroph	TAS [11]
MIGS-6	Habitat	Soil, root nodule, host	TAS [10]
MIGS-15	Biotic relationship	Free living, Symbiotic	TAS [10]
MIGS-14	Pathogenicity	None	NAS
	Biosafety level	1	TAS [20]
	Isolation	Root nodule of *Lotus corniculatus*	TAS [1]
MIGS-4	Geographic location	Lammermoor, Otago NZ	TAS [1]
MIGS-5	Nodule collection date	1993	TAS [1]
MIGS-4.1 MIGS-4.2	Latitude	-45.53	TAS [1]
	Longitude	169.9415	TAS [1]
MIGS-4.3	Depth	10 cm	IDA
MIGS-4.4	Altitude	885 meters	IDA

Evidence codes – IDA: Inferred from Direct Assay; TAS: Traceable Author Statement (i.e., a direct report exists in the literature); NAS: Non-traceable Author Statement (i.e., not directly observed for the living, isolated sample, but based on a generally accepted property for the species, or anecdotal evidence). These evidence codes are from the Gene Ontology project [21].

**Figure 2:**
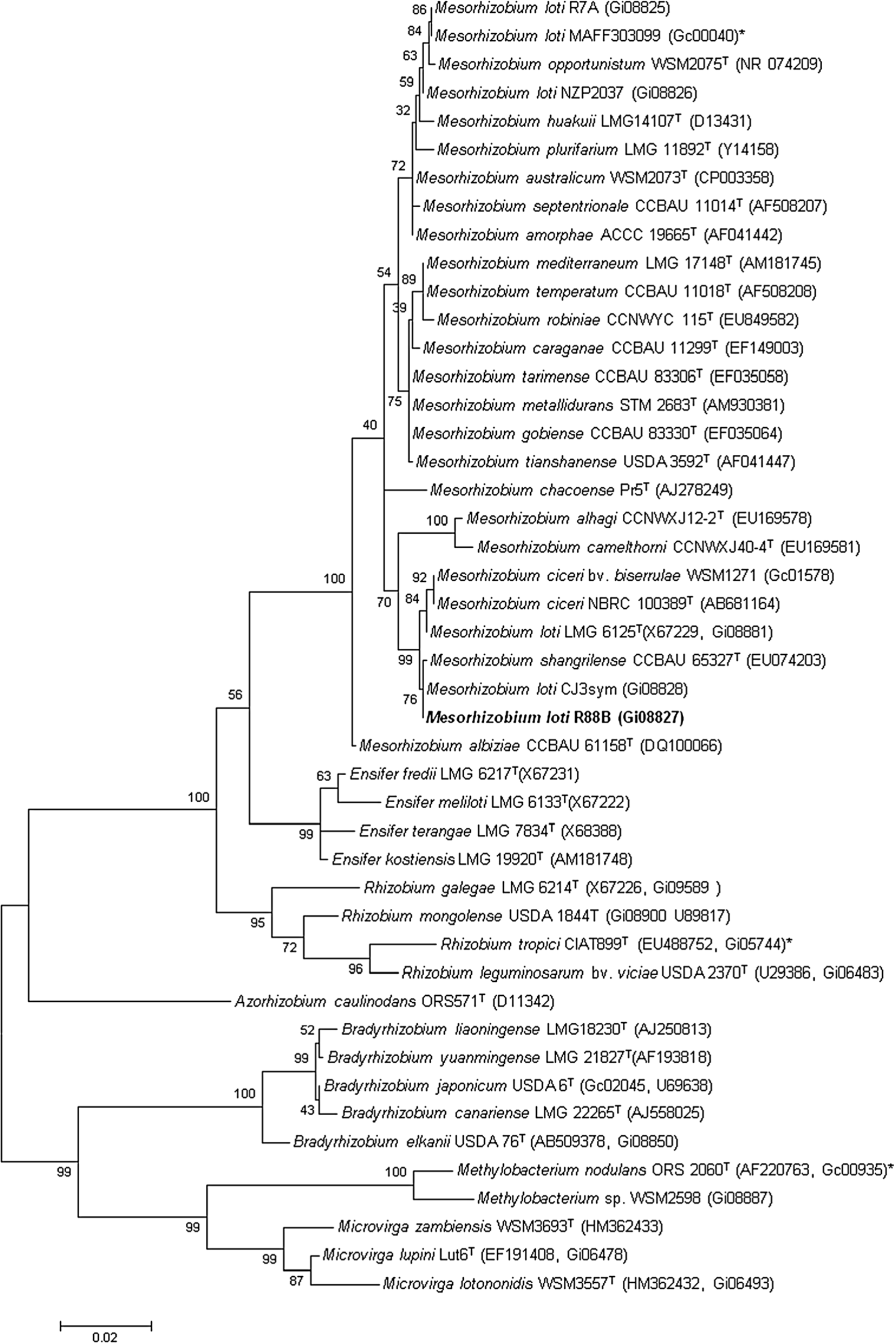
**Phylogenetic tree showing the relationships of *Mesorhizobium loti *R88B with other root nodule bacteria based on aligned sequences of the 16S rRNA gene (1,290 bp internal region).** All sites were informative and there were no gap-containing sites. Phylogenetic analyses were performed using MEGA [22], version 5. The tree was built using the Maximum-Likelihood method with the General Time Reversible model [23]. Bootstrap analysis [24] with 500 replicates was performed to assess the support of the clusters. Type strains are indicated with a superscript T. Brackets after the strain name contain a DNA database accession number and/or a GOLD ID (beginning with the prefix G) for a sequencing project registered in GOLD [25]. Published genomes are indicated with an asterisk.

Figure 2 shows the phylogenetic neighborhood of *M. loti* strain R88B in a 16S rRNA gene sequence based tree. This strain has 99.7% sequence identity (1364/1367 bp) at the 16S rRNA sequence level to the sequenced *M. australicum* WSM2073 (GOLD ID: Gc02468) and 99.6% 16S rRNA sequence (1362/1367 bp) identity to the fully sequenced *M. ciceri* bv. *biserrulae* WSM1271 (GOLD ID: Gc01578).

### Symbiotaxonomy

*M. loti* strain R88B was isolated from a stand of *L. corniculatus* bv. Goldie planted in 1986 at a field site which lacked naturalized rhizobia capable of nodulating the plant. The inoculum strain used was *M. loti* R7A (ICMP3153). The field site was an undeveloped tussock (*Festuca novae-zealandiae* and *Chionochloa rigida*) grassland located at an elevation of 885 m in Lammermoor, the Rocklands range, Otago, New Zealand. The soil was a dark brown silt loam with an acid pH (4.9) and a low (0.28%) total nitrogen content. Prior to establishment of the site, R88B likely existed as a soil saprophyte that lacked symbiotic DNA. Subsequent transfer of ICE*Ml*Sym^R7A^ from the donor strain R7A converted R88B into a symbiont and, hence enabled R88B to nodulate *L. corniculatus,* leading to the isolation of R88B when field sampling was performed in 1993. R88B forms effective nodules on *Lotus corniculatus*, but it has not been tested on any other *Lotus* species to date.

## Genome Sequencing Information

### Genome project history

This organism was selected for sequencing on the basis of its environmental and agricultural relevance to issues in global carbon cycling, alternative energy production, and biogeochemical importance, and is part of the Community Sequencing Program at the U.S. Department of Energy, Joint Genome Institute (JGI), which is focused on projects of relevance to agency missions. The genome project is deposited in the Genomes OnLine Database [25] and an improved-high-quality-draft genome sequence in IMG. Sequencing, finishing and annotation were performed by the JGI. A summary of the project information is shown in Table 2.

**Table 2:** **Genome sequencing project information for *Mesorhizobium loti *** **R88B**

**MIGS ID**	**Property**	**Term**
MIGS-31	Finishing quality	Improved-high-quality-draft
MIGS-28	Libraries used	Illumina Standard (short PE) and CLIP (long PE) libraries
MIGS-29	Sequencing platforms	Illumina HiSeq2000 technology
MIGS-31.2	Sequencing coverage	Illumina: 589×
MIGS-30	Assemblers	Velvet version 1.1.05; Allpaths-LG version r39750; phrap, version 4.24
MIGS-32	Gene calling method	Prodigal 1.4, GenePRIMP
	Genbank accession	JACE00000000
	Genbank date of release	12-OCT-2014
	GOLD ID	Gi08827
	NCBI project ID	76961
	Database: IMG	2512875024
	Project relevance	Symbiotic nitrogen fixation, agriculture

### Growth conditions and DNA isolation

*M. loti* strain R88B was grown to mid logarithmic phase in TY rich medium [26] on a gyratory shaker at 28°C at 250 rpm. DNA was isolated from 60 mL of cells using a CTAB (Cetyl trimethyl ammonium bromide) bacterial genomic DNA isolation method [27].

### Genome sequencing and assembly

The draft genome of *M. loti* R88B was generated at the DOE JGI using Illumina [28] technology. For this genome, we constructed and sequenced an Illumina short-insert paired-end library with an average insert size of 270 bp which generated 17,358,418 reads and an Illumina long-insert paired-end library with an average insert size of 4,146+/-2,487 bp which generated 10,904,934 reads totaling 4,240 Mbp of Illumina data (unpublished, Feng Chen). All general aspects of library construction and sequencing performed at the JGI can be found at the DOE Joint Genome Institute website [29].

The initial draft assembly contained 41 contigs in 9 scaffolds. The initial draft data were assembled with Allpaths, version 39750, and the consensus was computationally shredded into 10 Kbp overlapping fake reads (shreds). The Illumina draft data were also assembled with Velvet, version 1.1.05 [30], and the consensus sequences were computationally shredded into 1.5 Kbp overlapping fake reads (shreds). The Illumina draft data were assembled again with Velvet using the shreds from the first Velvet assembly to guide the next assembly. The consensus from the second VELVET assembly was shredded into 1.5 Kbp overlapping fake reads. The fake reads from the Allpaths assembly and both Velvet assemblies and a subset of the Illumina CLIP paired-end reads were assembled using parallel phrap, version 4.24 (High Performance Software, LLC). Possible mis-assemblies were corrected with manual editing in Consed [31-33]. Gap closure was accomplished using repeat resolution software (Wei Gu, unpublished), and sequencing of bridging PCR fragments with Sanger technology. For improved high quality draft, one round of manual/wet lab finishing was completed. A total of 23 additional sequencing reactions were completed to close gaps and to raise the quality of the final sequence. The total (“estimated size” for unfinished) size of the genome is 7.2 Mbp and the final assembly is based on 4,240 Mbp of Illumina draft data, which provided an average 589× coverage of the genome.

### Genome annotation

Genes were identified using Prodigal [34] as part of the Oak Ridge National Laboratory genome annotation pipeline, followed by a round of manual curation using the JGI GenePrimp pipeline [35]. The predicted CDSs were translated and used to search the National Center for Biotechnology Information (NCBI) nonredundant database, UniProt, TIGRFam, Pfam, PRIAM, KEGG, COG, and InterPro databases. These data sources were combined to assert a product description for each predicted protein. Non-coding genes and miscellaneous features were predicted using tRNAscan-SE [36], RNAMMer [37], Rfam [38], TMHMM [39], and SignalP [40]. Additional gene prediction analyses and functional annotation were performed within the Integrated Microbial Genomes (IMG-ER) platform [41].

## Genome properties

**Figure 3:**
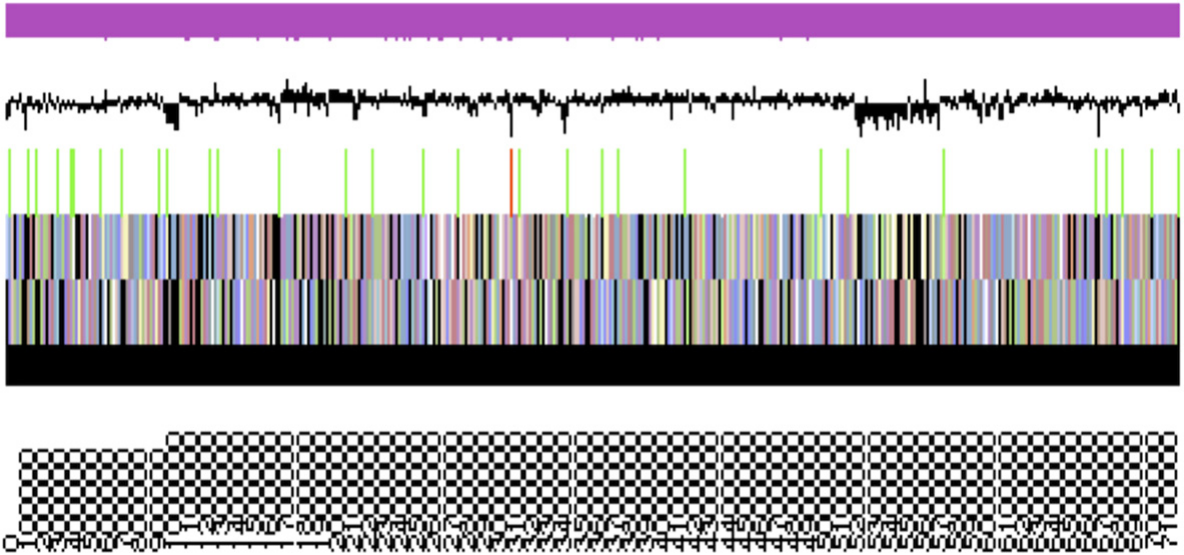
**Graphical map of the single scaffold of *Mesorhizobium loti *R88B.** From bottom to the top: Genes on forward strand (color by COG categories as denoted by the IMG platform), Genes on reverse strand (color by COG categories), RNA genes (tRNAs green, sRNAs red, other RNAs black), GC content, GC skew.

The genome is 7,195,110 nucleotides with 62.37% GC content (Table 3) and is comprised of a single scaffold and no plasmids. From a total of 7,016 genes, 6,950 were protein encoding and 66 RNA-only encoding genes. Within the genome, 189 pseudogenes were also identified. The majority of genes (79.13%) were assigned a putative function whilst the remaining genes were annotated as hypothetical. The distribution of genes into COGs functional categories is presented in Table 4 and Figure 3.

**Table 3:** **Genome statistics for *Mesorhizobium loti *** **R88B**

**Attribute**	**Value**	**% of total**
Genome size (bp)	7,195,110	100.00
DNA coding region (bp)	6,308,527	87.68
DNA G + C content (bp)	4,487,516	62.37
Number of scaffolds	1	
Number of contigs	14	
Total genes	7,016	100.00
RNA genes	66	0.94
rRNA operons	2*	
Protein-coding genes	6,950	99.06
Genes with function prediction	5,552	79.13
Genes assigned to COGs	5,511	78.55
Genes assigned Pfam domains	5,800	82.67
Genes with signal peptides	651	9.28
Genes coding transmembrane proteins	1,669	23.79

*2 copies if 5S, 2 copies of 16S and 2 copies of 23S rRNA.

**Table 4:** **Number of protein coding genes of *Mesorhizobium loti *** **R88B associated with the general COG functional categories**

**Code**	**Value**	**% age**	**COG category**
J	202	3.29	Translation, ribosomal structure and biogenesis
A	1	0.02	RNA processing and modification
K	572	9.32	Transcription
L	181	2.95	Replication, recombination and repair
B	5	0.08	Chromatin structure and dynamics
D	33	0.54	Cell cycle control, mitosis and meiosis
Y	0	0.00	Nuclear structure
V	63	1.03	Defense mechanisms
T	238	3.88	Signal transduction mechanisms
M	330	5.38	Cell wall/membrane biogenesis
N	43	0.70	Cell motility
Z	1	0.02	Cytoskeleton
W	1	0.02	Extracellular structures
U	122	1.99	Intracellular trafficking and secretion
O	188	3.06	Posttranslational modification, protein turnover, chaperones
C	338	5.51	Energy production conversion
G	567	9.24	Carbohydrate transport and metabolism
E	751	12.24	Amino acid transport metabolism
F	90	1.47	Nucleotide transport and metabolism
H	230	3.75	Coenzyme transport and metabolism
I	257	4.19	Lipid transport and metabolism
P	272	4.43	Inorganic ion transport and metabolism
Q	204	3.32	Secondary metabolite biosynthesis, transport and catabolism
R	830	13.52	General function prediction only
S	619	10.08	Function unknown
-	1,505	21.45	Not in COGS

## Conclusion

The *M. loti* strain R88B genome consists of a single chromosome of 7.2 Mb predicted to encode 7,016 genes. The sequencing was completed to the stage where a single scaffold comprising 14 contigs was obtained. *M. loti* strain R88B was isolated in New Zealand as a strain that gained symbiotic ability through receiving the *M. loti* strain R7A symbiosis island (now referred to as ICE*Ml*Sym^R7A^) in the field environment [1-3]. On the basis of 16S rRNA gene sequence similarity, strains able to nodulate *Lotus* species that have been examined to date appear to fall into two clusters (Figure 2). R88B is more closely related to *M. loti* strains LMG 6125 and CJ3Sym and *M. ciceri* strains NBRC 100389 and bv. *biserrulae* WSM1271, than to strains R7A, NZP2037 and MAFF303099 from which its 16SrRNA gene differs by over 20 nucleotides. It is clear that within the mesorhizobia the degree of 16S rRNA gene sequence similarity observed between strains does not necessarily reflect host range. Strain R88B was also shown to contain at least two further regions of acquired DNA adjacent to ICE*Ml*sym^R7A^ that were likely present prior to arrival of ICE*Ml*Sym^R7A^, indicating that tandem, sequential acquisition of elements that provide adaptive traits occurred at the phe-tRNA locus. One of these elements, ICE*Ml*Adh^R88B^, encoded an adhesin and *tra* genes required for mobilization *in trans* by another conjugative element. The other was a region containing *fhu* genes involved in iron acquisition that was found to be one of three genomic regions required for utilization of the siderophore ferrichrome [6].

## Competing interests

The authors declare that they have no competing interests.

## Authors’ contributions

JS and CR supplied the strain and background information for this project and helped WR write the paper, TR supplied DNA to JGI and performed all imaging, WR coordinated the project and all other authors were involved in either sequencing the genome and/or editing the paper. All authors read and approved the final manuscript.
